# Global trends and partial forecast of adverse effects of medical treatment from 1990 to 2019: an epidemiological analysis based on the global burden of disease study 2019

**DOI:** 10.1186/s12889-023-17560-0

**Published:** 2024-01-25

**Authors:** Xin Kong, Xufeng Tao, Lu Li, Xinya Zhao, Jiaqi Ren, Shilei Yang, Xuyang Chen, Hong Xiang, Guoyu Wu, Yunming Li, Deshi Dong

**Affiliations:** 1 Department of Pharmacy, First Affiliated Hospital of Dalian Medical University, Dalian, 116011 China; 2 School of pharmacy, Dalian Medical University, Dalian, 116044 China; 3 Laboratory of Integrative Medicine, First Affiliated Hospital of Dalian Medical University, Dalian, 116011 China

**Keywords:** Adverse effects of medical treatment, Medical errors, Patient safety, Health care system, Global burden of Disease

## Abstract

**Background:**

The possibility of adverse effects of medical treatment (AEMT) is increasing worldwide, but little is known about AEMT in China. This study analyzed the health burden of AEMT in China in recent years through the Global Burden of Disease Study (GBD) 2019 and compared it with the worldwide average level and those in different sociodemographic index (SDI) regions.

**Methods:**

We calculated the age-standardized rate (ASR) of deaths, disability-adjusted life years (DALYs), years of life lost (YLLs), years lived with disability (YLDs), incidence and prevalence attributed to AEMT in China, worldwide and countries with different sociodemographic indices during 1990–2019 using the latest data and methods from the GBD 2019.

**Results:**

From 1990 to 2019, the global age-standardized death rate (ASDR), DALYs, and YLLs for AEMT showed a significant downward trend and were negatively associated with the SDI. By 2040, the ASDR is expected to reach approximately 1.58 (95% UI: 1.33–1.80). From 1990 to 2019, there was no significant change in the global incidence of AEMT. The occurrence of AEMT was related to sex, and the incidence of AEMT was greater among females. In addition, the incidence of AEMT-related injuries and burdens, such as ASR of DALYs, ASR of YLLs and ASR of YLDs, was greater among women than among men. Very old and very young people were more likely to be exposed to AEMT.

**Conclusions:**

From 1990 to 2019, progress was made worldwide in reducing the harm caused by AEMT. However, the incidence and prevalence of AEMT did not change significantly overall during this period. Therefore, the health sector should pay more attention to AEMT and take effective measures to reduce AEMT.

## Introduction

The adverse effects of medical treatment (AEMT) are commonly defined as “unintentional injuries during medical activities that affect a patient’s diagnosis, increase the patient’s pain and burden, and cause serious long-term irreversible consequences or death” [1]. AEMT are a result of a medical condition occurring widely in health care settings rather than the patient’s underlying disease [2]. In 1984, the Harvard Medical Practice Study was the first to provide an estimate of medical injuries in U.S. hospitals, and medical errors were estimated to be the third leading cause of death in the United States [1, 3]. In the 1980s, adverse reaction monitoring began in China, and currently, there are three main medical adverse event reporting systems. The adverse drug reaction monitoring system, gross medical negligence and medical malpractice reporting system, and patient safety (adverse event) reporting system are used [4]. Progress was made in reducing deaths from AEMT in the UK between 1990 and 2013, but unfortunately, the incidence of AEMT has not changed [5]. The occurrence of AEMT is common worldwide; fortunately, most adverse events are preventable [6]. Therefore, an increasing number of countries and regions have begun to attach importance to AEMT, which led to a key strategy developed by the World Health Organization (WHO) on AEMT that aims to halve the global incidence of AEMT by 2022 [7]. However, in light of physicians’ fears of negative consequences and lack of knowledge about AEMT, little is known about the levels, trends, and patterns of AEMT in hospital settings in China over time [8].

AEMT are a global health problem. Although the detection methods and reporting systems for AEMT have improved in recent years, there are still many worries and challenges [9]. The Global Burden of Disease Study (GBD) quantifies the global health losses caused by different diseases, injuries and risk factors. In addition, the GBD can use time, geographic location, age and sex to calculate the course of health loss caused by different causes [10]. Based on the GBD 2019, this study described the changing trend of the ASR of the incidence, prevalence, deaths, DALYs, YLDs and YLLs due to AEMT in China from 1990 to 2019, as well as forecasts of possible trends in the future. Although GBD data are estimated, this study is conducive to strengthening the public’s understanding of AEMT.

## Methods

### GBD 2019

The Global Study on Diseases, Injuries and Risk Factors is a systematic and collaborative global database (https://vizhub.healthdata.org/gbd-results/) that was established to quantify the health losses caused by hundreds of diseases from 1990 to 2019 according to age, sex and geographic location [11]. The GBD is based on multiple data sources (epidemiological studies, health registries, official statistics, hospital data, etc.) and applies highly sophisticated models to fill in data gaps to make the most informed estimates. Cause-specific death rates and cause fractions were calculated using the cause of death ensemble model and spatiotemporal Gaussian process regression. Cause-specific deaths were adjusted for to match the total all-cause deaths calculated as part of the GBD population, fertility, and mortality estimates [12, 13]. The list of causes in the GBD is detailed and regular due to the International Classification of Diseases (ICD) codes used for statistics [13]. We used the ICD-10 to define AEMT and then retrieved and analyzed epidemiological data attributed to AEMT from the latest GBD 2019. The forecast data for the ASDR and ASR of YLLs were obtained from the following website: https://vizhub.healthdata.org/gbd-foresight/.

### Incidence and prevalence

The incidence was defined as the number of new cases of AEMT in a given region or country at a given time (one year) divided by the population of that region or country. The main distinction between prevalence and incidence is that the incidence emphasizes the frequency of new cases in a given population within a given period of time. The prevalence is a better indicator of the long-term occurrence of certain diseases and their impact on public health.

### Years of Life Lost (YLLs), Years Lived with Disability (YLDs) and Disability-Adjusted Life Years (DALYs)

The guiding principle of the GBD is a comprehensive assessment of health loss due to death and disability, with disability defined as any deviation from full health. The Global Health Data Exchange (GHDx, http://ghdx.healthdata.org/) is the world’s largest health data repository. DisMod-MR 2.1 is a Bayesian meta-regression tool that was our primary method for analyzing nonmortality data [14]. YLLs are equal to the difference in standard life expectancy minus age at death, and YLDs are the number of years lived with disability. DisMod-MR 2.1 or meta-analysis is used to summarize the proportion of patients with each sequela or from patient series analysis. The YLD estimates are obtained by multiplying the prevalence of sequelae for each disease by an appropriate disability weight factor. YLLs are estimated by multiplying the number of deaths by the standard ideal remaining life expectancy at the time of death [15]. DALYs, a sum of YLLs and YLDs, use the total health loss of the population as a variable to estimate the total burden of AEMT.

### Death rates

The death rate is the proportion of AEMT deaths per 100,000 people and is determined by the number of deaths caused by AEMT per year. Age-standardized death rates (ASDRs) can be used to effectively address the differences in mortality rates caused by population size and age structure in different regions. The WHO has modeled cause-of-death clusters for ASDRs under various conditions. The GBD model is used as a standardized tool for mortality causation analysis to obtain the ASDR across age, sex, geography, and year [16].

### Sociodemographic index

The sociodemographic index (SDI) is a comprehensive indicator of the development status of countries in terms of overall national wealth. The SDI is a comprehensive evaluation of the average education level of the population aged 15 years and older, the average per capita income under a lagged distribution, and the total fertility rate of the population under 25 years old. The SDI classifies 195 countries and regions into five grades: low SDI, low middle SDI, middle SDI, high middle SDI and high SDI. Integrated analysis of the disease burden and SDI can better reveal the link between a specific disease and the development status of a country [17].

### Statistical analysis

The GBD 2019 was used not only to calculate the ASR of various indicators for different ages, sexes and years but also to estimate the 95% uncertainty interval (UI) to quantify the total burden of adverse medical events between 1990 and 2019. The annual rate of change (ARC) can more directly display the temporal change trend of various data. The age group and the sex ratio can make the trend more visible. All the data were analyzed using GraphPad Prism 8.0.

## Results

### ASDRs of AEMT in China, worldwide and in other countries and regions with different SDI levels, 1990–2019

**Fig. 1:**
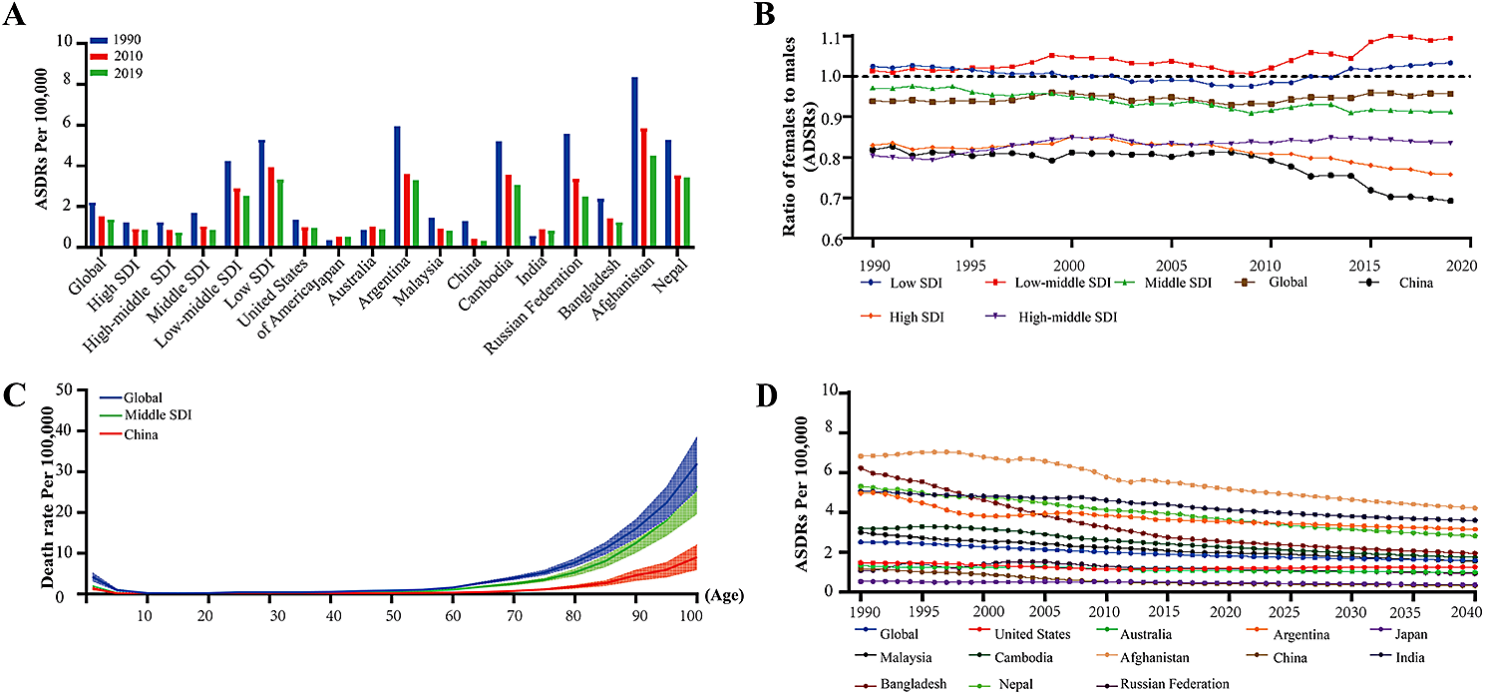
Trends in ASDRs due to AEMT in China, in different SDI regions and globally, disaggregated by sex and age, 1990–2019. **(A)** ASDRs due to AEMT in China, SDI regions and globally in 1990, 2010 and 2019. **(B)** The difference and change trend of the sex ratio (female to male) of ASDRs due to AEMT in China from 1990 to 2019. **(C)** The age difference in the ASDR due to AEMT between China and the SDI regions. **(D)** The projected trend based on the change in the ASDR due to AEMT from 1990 to 2019, projected to 2040. ASDR, age-standardized death rate; AEMT, adverse effects of medical treatment; SDI, sociodemographic index

As shown in Table 1, there was a clear negative correlation between the ASDRs due to AEMT and the SDI levels from 1990 to 2019. From 1990 to 2019, the global ASDRs caused by AEMT showed a downward trend; China had an ARC of -0.76 (95% UI: -0.82 to -0.61) (Fig. 1A). The ratio of males to females in AEMT-related ASDRs was correlated with the SDI. In low-SDI areas, the ASDRs of AEMT were approximately the same for males and females. However, in low-middle-SDI regions, women had higher ASDRs than men did, and in other SDI areas, the ASDRs were greater for men than for women. From a sex perspective, the female-to-male ratio of ASDRs caused by AEMT in China has shown a significant downward trend in the past decade, and the ratio dropped to 0.64 in 2019.In 2019, the global ratio of males to females in ASDR due to AEMT was approximately 0.95 (Fig. 1B). Figure 1C shows the age distribution of the death rate due to AEMT in China, middle SDI regions and worldwide, and all of them showed similar trends. Mortality from AEMT showed a slightly higher in the early years of life, with a low death rate in the 10–60 age group, while the death rate of elderly individuals (over 60 years old) increased with age. China’s death rate attributed to AEMT was lower than that worldwide in any age group. The ASDR due to AEMT is expected to decline steadily in all regions by 2040. The global ASDRs for AEMT are predicted to be 1.67 (95% UI: 1.43–1.86) in 2030 and 1.58 (95% UI: 1.33–1.80) in 2040, and they are estimated to be 0.36 (95% UI: 0.31–0.48) in 2030 and 0.31 (95% UI: 0.26–0.41) in 2040 in China. Notably, countries in the low-SDI and middle-SDI regions still had high ASDRs (Fig. 1D).

**Table 1:** ASDRs (per 100,000 people) and their relative changes due to AEMT for countries and regions with different SDI levels in 1990, 2010 and 2019

Location	ASDR (95% UI)			ARC (95% UI)		
	1990	2010	2019	1990–2010	2010–2019	1990–2019
Global	2.20 (1.75–2.55)	1.54 (1.29–1.74)	1.36 (1.15–1.51)	-0.30 (-0.34 to -0.23)	-0.12 (-0.19 to -0.04)	-0.38 (-0.45 to -0.29)
High SDI	1.23 (1.02–1.30)	0.89 (0.79–0.97)	0.86 (0.74–0.92)	-0.27 (-0.31 to -0.17)	-0.04 (-0.08 to 0.00)	-0.30 (-0.34 to -0.21)
High-middle SDI	1.22 (1.04–1.31)	0.83 (0.72–0.88)	0.72 (0.61–0.78)	-0.32 (-0.37 to -0.25)	-0.13 (-0.18 to -0.09)	-0.41 (-0.46 to -0.35)
Middle SDI	1.69 (1.31–1.90)	1.02 (0.89–1.19)	0.86 (0.74-1.00)	-0.39 (-0.47 to -0.27)	-0.16 (-0.23 to -0.08)	-0.49 (-0.59 to -0.37)
Low-middle SDI	4.25 (3.02–5.34)	2.88 (2.20–3.30)	2.54 (1.99–2.96)	-0.32 (-0.41 to -0.23)	-0.12 (-0.27 to 0.02)	-0.40 (-0.55 to -0.25)
Low SDI	5.27 (3.52–8.35)	3.92 (2.79–6.20)	3.31 (2.43–4.81)	-0.26 (-0.31 to -0.18)	-0.16 (-0.24 to -0.05)	-0.37 (-0.46 to -0.24)
United States	1.37 (1.18–1.49)	0.97 (0.89–1.22)	0.95 (0.87–1.18)	-0.29 (-0.33 to -0.09)	-0.02 (-0.05 to 0.01)	-0.31 (-0.34 to -0.13)
Japan	0.36 (0.33–0.48)	0.52 (0.42–0.56)	0.50 (0.40–0.55)	0.46 (-0.08 to 0.59)	-0.04 (-0.10 to 0.00)	0.40 (-0.11 to 0.55)
Australia	0.83 (0.74–0.96)	1.02 (0.72–1.12)	0.88 (0.68–0.98)	0.22 (-0.09 to 0.35)	-0.14 (-0.21 to -0.05)	0.05 (-0.15 to 0.17)
Argentina	5.96 (4.40–6.41)	3.58 (3.20–4.03)	3.29 (2.90–3.69)	-0.40 (-0.45 to -0.10)	-0.08 (-0.14 to -0.01)	-0.45 (-0.50 to -0.16)
Malaysia	1.47 (1.00-1.99)	0.93 (0.72–1.06)	0.80 (0.60–1.03)	-0.37 (-0.54 to -0.18)	-0.14 (-0.34 to 0.07)	-0.46 (-0.66 to -0.21)
China	1.28 (0.87–1.49)	0.42 (0.37–0.55)	0.31 (0.25–0.40)	-0.67 (-0.74 to -0.48)	-0.28 (-0.37 to -0.18)	-0.76 (-0.82 to -0.61)
India	5.21 (3.68–6.40)	3.54 (2.62–4.06)	3.05 (2.28–3.74)	-0.32 (-0.40 to -0.23)	-0.14 (-0.32 to 0.04)	-0.41 (-0.57 to -0.25)
Russian Federation	0.55 (0.49–0.89)	0.88 (0.68–0.92)	0.81 (0.56–0.95)	0.59 (-0.14 to 0.86)	-0.08 (-0.22 to 0.05)	0.46 (-0.22 to 0.85)
Bangladesh	5.56 (3.76–8.36)	3.37 (2.43–5.08)	2.48 (1.77–3.52)	-0.39 (-0.48 to -0.26)	-0.27 (-0.40 to -0.10)	-0.55 (-0.66 to -0.40)
Cambodia	2.38 (1.64–3.58)	1.42 (0.96–2.25)	1.22 (0.85–1.97)	-0.40 (-0.53 to -0.26)	-0.14 (-0.28 to 0.01)	-0.49 (-0.61 to -0.33)
Afghanistan	8.36 (3.47–14.64)	5.84 (2.65–10.52)	4.51 (2.23–7.59)	-0.30 (-0.46 to -0.10)	-0.23 (-0.35 to -0.05)	-0.46 (-0.59 to -0.27)
Nepal	5.27 (3.34–8.82)	3.52 (2.56–5.35)	3.43 (2.46–4.90)	-0.33 (-0.48 to -0.14)	-0.03 (-0.21 to 0.18)	-0.35 (-0.54 to -0.10)

a: ASDR, age-standardized death rate; AEMT, adverse effects of medical treatment; SDI, sociodemographic index; ARC, annual rate of change; UI, uncertainty interval

### DALY rates due to AEMT in China, worldwide and in other countries and regions with different SDI levels, 1990–2019

**Fig. 2:**
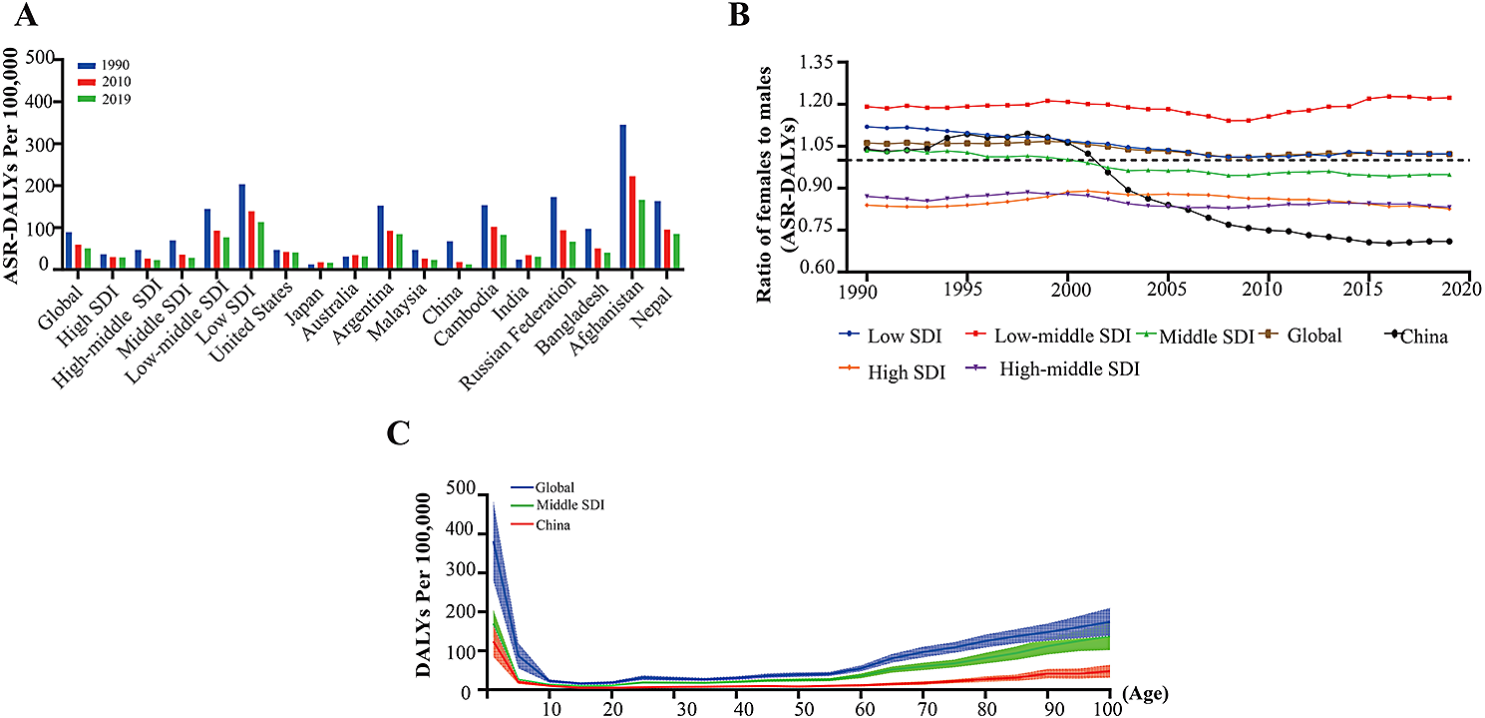
Trends in ASR-DALYs caused by AEMT in China and other regions with different SDIs disaggregated by sex and age, 1990–2019. **(A)** ASR-DALYs due to AEMT in China, the SDI region, and globally in 1990, 2010 and 2019. **(B)** Sex differences and trends in ASR-DALYs induced by AEMT in China during 1990–2019. **(C)** Age difference in ASR-DALYs caused by AEMT between China and regions with SDI levels worldwide. ASR, age-standardized rate; DALYs, disability-adjusted life years; AEMT, adverse effects of medical treatment; SDI, sociodemographic index

The ASR of DALYs caused by AEMT exhibited a significant downward trend worldwide during 1990–2019. During the study period, the ASR of DALYs for AEMT in China decreased most significantly, from 67.65 (95% UI: 44.17–81.67) in 1990 to 12.33 (95% UI: 10.42–15.77) in 2019, with an ARC of -0.82 (95% UI: -0.86 to -0.70). Although most countries showed a downward trend for AEMT-related ASRs of DALYs, both Japan and Russia presented a trend of initial growth and then a decline but an overall rise. In addition, the SDI strongly influenced the ASRs of DALYs (Table 2; Fig. 2A).

**Table 2:** ASR-DALYs (per 100,000 population) and their relative changes due to AEMT for countries and regions with different SDI levels in 1990, 2010 and 2019

Region	ASR-DALYs (95% UI)			ARC (95% UI)		
	1990	2010	2019	1990–2010	2010–2019	1990–2019
Global	89.04 (67.34-102.32)	59.68 (48.75–67.43)	50.44 (41.60-57.68)	-0.33 (-0.38 to -0.25)	-0.15 (-0.22 to -0.08)	-0.43 (-0.50 to -0.34)
High SDI	36.69 (32.06–40.32)	29.73 (26.40-33.53)	28.87 (25.37–32.79)	-0.19 (-0.24 to -0.10)	-0.03 (-0.06 to 0.00)	-0.21 (-0.27 to -0.13)
High-middle SDI	46.70 (38.46–51.92)	26.56 (24.29–28.60)	22.59 (19.82–24.81)	-0.43 (-0.48 to -0.34)	-0.15 (-0.20 to -0.10)	-0.52 (-0.57 to -0.43)
Middle SDI	69.58 (52.40-78.29)	35.64 (30.91–40.78)	28.32 (24.76–32.43)	-0.49 (-0.54 to -0.38)	-0.21 (-0.27 to -0.14)	-0.59 (-0.65 to -0.49)
Low-middle SDI	144.77 (104.48-168.81)	92.93 (71.26-103.76)	76.93 (60.33–89.46)	-0.36 (-0.41 to -0.27)	-0.17 (-0.28 to -0.07)	-0.47 (-0.56 to -0.36)
Low SDI	203.47 (143.70-270.03)	139.09 (102.26-193.92)	113.35 (85.02-153.98)	-0.32 (-0.39 to -0.22)	-0.19 (-0.26 to -0.09)	-0.44 (-0.52 to -0.33)
United States	46.80 (41.43–53.03)	42.04 (35.84–50.98)	40.86 (34.75–49.51)	-0.10 (-0.17 to 0.01)	-0.03 (-0.05 to 0.00)	-0.13 (-0.19 to -0.02)
Japan	12.29 (11.38–15.15)	17.42 (13.08–18.51)	16.31 (12.46–17.70)	0.42 (-0.09 to 0.54)	-0.06 (-0.12 to -0.02)	0.33 (-0.12 to 0.47)
Australia	31.24 (26.60-37.33)	34.54 (28.31–40.93)	31.52 (25.94–37.86)	0.11 (-0.04 to 0.18)	-0.09 (-0.14 to -0.03)	0.01 (-0.10 to 0.08)
Argentina	152.57 (118.75-162.29)	92.60 (85.93-106.14)	84.50 (77.10-97.85)	-0.39 (-0.44 to -0.09)	-0.09 (-0.15 to -0.02)	-0.45 (-0.50 to -0.13)
Malaysia	46.85 (33.00-59.51)	26.62 (21.74–30.07)	23.10 (17.56–29.64)	-0.43(-0.55to-0.28)	-0.13 (-0.34 to 0.10)	-0.51 (-0.68 to -0.30)
China	67.65 (44.17–81.67)	18.11 (16.01–22.69)	12.33 (10.42–15.77)	-0.73 (-0.79 to -0.57)	-0.32 (-0.40 to -0.24)	-0.82 (-0.86 to -0.70)
India	153.37 (108.34-177.69)	101.82 (75.86-115.01)	82.65 (61.82–99.37)	-0.34 (-0.40 to -0.25)	-0.19 (-0.33 to -0.05)	-0.46 (-0.57 to -0.34)
Russian Federation	23.86 (21.36–33.04)	34.65 (27.28–36.89)	30.84 (22.53–35.75)	0.45 (-0.11 to 0.64)	-0.11 (-0.23 to 0.02)	0.29 (-0.19 to 0.56)
Bangladesh	173.01 (122.97-232.54)	93.68 (68.90-139.28)	66.68 (47.48–98.25)	-0.46 (-0.57 to -0.31)	-0.29 (-0.40 to -0.15)	-0.61 (-0.71 to -0.49)
Cambodia	97.22 (72.18-144.44)	50.62 (35.25–79.75)	40.30 (27.96–63.56)	-0.48 (-0.60 to -0.34)	-0.20 (-0.34 to -0.04)	-0.59 (-0.69 to -0.46)
Afghanistan	345.33 (154.20-529.52)	222.40 (108.17-362.81)	166.31 (85.34-263.08)	-0.36 (-0.50 to -0.16)	-0.25 (-0.37 to -0.09)	-0.52 (-0.63 to -0.34)
Nepal	163.36 (110.41-242.97)	95.43 (68.66-143.52)	85.07 (61.10-123.72)	-0.42 (-0.54 to -0.27)	-0.11 (-0.25 to 0.07)	-0.48 (-0.60 to -0.33)

a: ASR-DALYs, age-standardized disability-adjusted life years; AEMT, adverse effects of medical treatment; SDI, sociodemographic index; ARC, annual rate of change; UI, uncertainty interval

In 2019, the global ASR of DALYs due to AEMT was estimated to be slightly greater for women than for men. In China, the ASR of DALYs caused by AEMT changed significantly in terms of sex from 1990 to 2019. Specifically, the female-to-male ratio of the ASR of DALYs in China showed an upward then downward trend, 1998 as the inflection point. Since 2002, there have been more ASRs of DALYs caused by AEMT among Chinese men than among women, and the sex-specific gap has gradually widened (Fig. 2B). In addition, the change trends of DALY rates in China, middle SDI regions and worldwide were roughly the same. Overall, the DALY rate of AEMT was particularly high at early ages and gradually increased for older people (over 65 years of age) after a trough from 10 to 60 years of age (Fig. 2C).

### Rates of YLLs and YLDs due to AEMT in China, worldwide and in other countries and regions with different SDI levels, 1990–2019

**Fig. 3:**
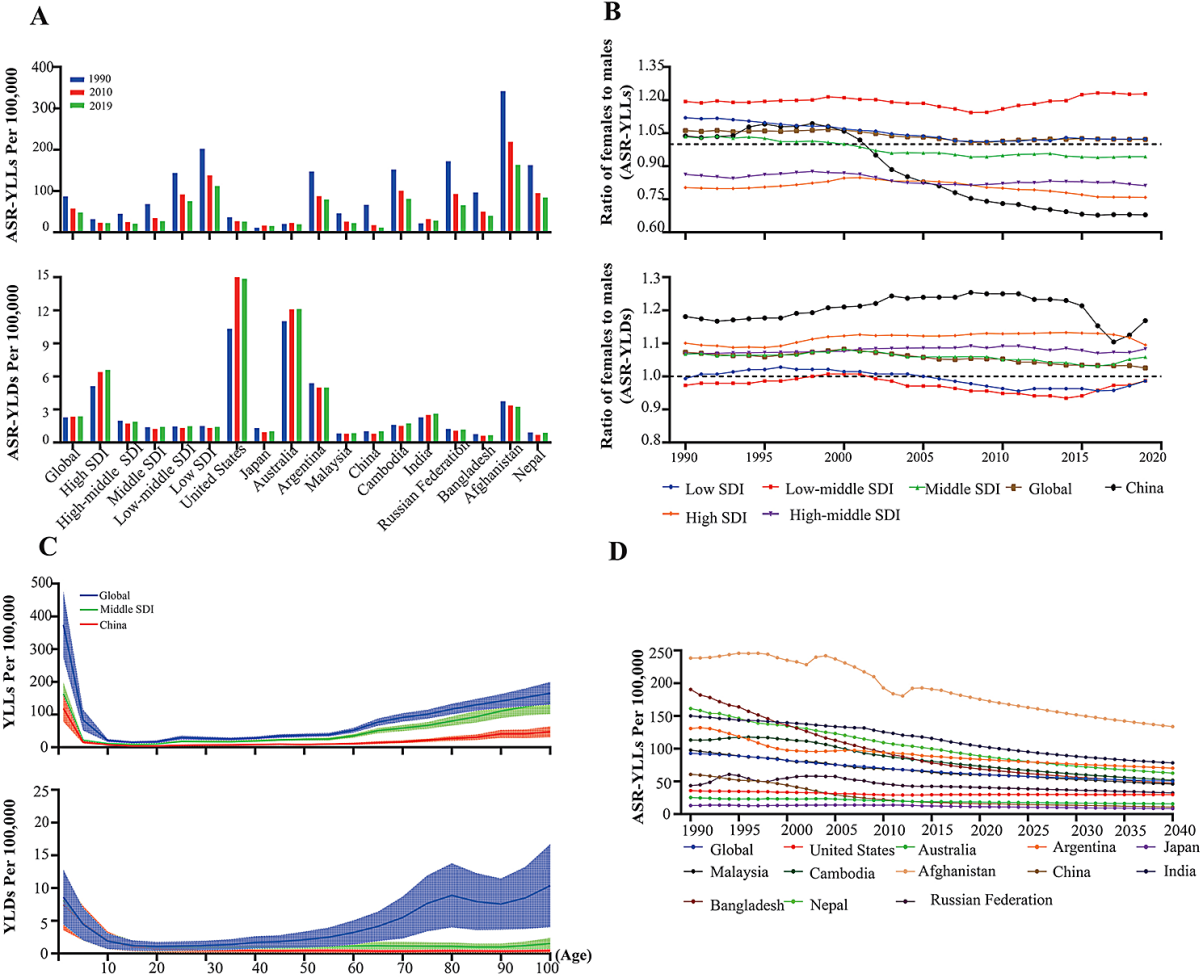
Comparison of YLL and YLD values caused by AEMT in China and in different SDI regions from 1990 to 2019. **(A)** Comparison of the ASRs of YLLs and YLDs caused by AEMT in China and SDI regions and globally in 1990, 2010 and 2019. **(B)** Sex differences and trends in ASR-YLLs and ASR-YLDs caused by AEMT from 1990 to 2019. **(C)** Age differences in ASR-YLLs and ASR-YLDs caused by AEMT between China and the SDI region. **(D)** Projected trends based on changes in the global ASR of YLLs due to AEMT from 1990 to 2019, projected to 2040. ASR, age-standardized rate; YLLs, years of life lost; YLDs, years lived with disability; AEMT, adverse effects of medical treatment; SDI, social demographic index

As shown in Tables 3 and 4; Fig. 3A, from 1990 to 2019, the global ASR-YLLs due to AEMT showed a significant downward trend, and the global ASR-YLDs was almost unchanged. The ASR-YLLs attributed to AEMT was also correlated with the SDI. In general, the higher the SDI was, the lower the ASR-YLLs. Most countries presented a clear downward trend in AEMT related ASR-YLLs; of those, China topped the list with an ARC of -0.83 (95% UI: -0.87 to -0.72).

**Table 3:** ASR-YLDs (per 100,000 population) and their relative changes in countries and regions with different SDI levels in 1990, 2010 and 2019 due to AEMT.

Region	ASR-YLDs (95% UI)			ARC (95% UI)		
	1990	2010	2019	1990–2010	2010–2019	1990–2019
Global	2.28 (1.44–3.39)	2.34 (1.49–3.47)	2.37 (1.50–3.54)	0.03 (-0.01 to 0.07)	0.01 (-0.02 to 0.05)	0.04 (0.02–0.07)
High SDI	5.11 (3.23–7.60)	6.40 (4.04–9.47)	6.58 (4.13–9.79)	0.25 (0.17–0.34)	0.03 (-0.01 to 0.07)	0.29 (0.24–0.36)
High-middle SDI	1.98 (1.22–3.01)	1.72 (1.07–2.59)	1.89 (1.17–2.90)	-0.13 (-0.15 to -0.11)	0.10 (0.06–0.13)	-0.05 (-0.07 to -0.02)
Middle SDI	1.39 (0.85–2.15)	1.22 (0.75–1.87)	1.41 (0.86–2.19)	-0.12 (-0.14 to -0.11)	0.16 (0.13–0.19)	0.02 (-0.01 to 0.05)
Low-middle SDI	1.46 (0.92–2.17)	1.33 (0.83–1.99)	1.48 (0.92–2.26)	-0.09 (-0.10 to -0.07)	0.11 (0.08–0.15)	0.02 (-0.02 to 0.05)
Low SDI	1.49 (0.94–2.22)	1.33 (0.84–1.96)	1.41 (0.89–2.11)	-0.11 (-0.13 to -0.10)	0.06 (0.03–0.09)	-0.06 (-0.08 to -0.03)
United States	10.32 (6.56–15.37)	15.00 (9.38–22.12)	14.85 (9.33–22.33)	0.45 (0.33–0.58)	-0.01 (-0.06 to 0.04)	0.44 (0.35–0.54)
Japan	1.32 (0.80–2.05)	0.93 (0.58–1.39)	1.02 (0.63–1.54)	-0.29 (-0.36 to -0.21)	0.09 (0.04–0.14)	-0.23 (-0.28 to -0.17)
Australia	11.00 (6.87–16.47)	12.09 (7.57–17.85)	12.11 (7.64–18.07)	0.10 (0.04–0.16)	0.00 (-0.05 to 0.05)	0.10 (0.05–0.15)
Argentina	5.38 (3.45–7.97)	4.98 (3.17–7.36)	4.98 (3.17–7.32)	-0.08 (-0.12 to -0.02)	0.00 (-0.05 to 0.06)	-0.08 (-0.13 to -0.02)
Malaysia	0.82 (0.49–1.29)	0.80 (0.47–1.27)	0.84 (0.50–1.34)	-0.02 (-0.08 to 0.03)	0.06 (-0.01 to 0.13)	0.03 (-0.04 to 0.12)
China	1.02 (0.61–1.62)	0.80 (0.47–1.28)	1.02 (0.59–1.65)	-0.21 (-0.25 to -0.18)	0.27 (0.23–0.32)	0.00 (-0.06 to 0.07)
India	1.60 (1.01–2.39)	1.50 (0.94–2.24)	1.74 (1.09–2.64)	-0.06 (-0.08 to -0.04)	0.16 (0.12–0.20)	0.09 (0.05–0.14)
Russian Federation	2.28 (1.41–3.45)	2.51 (1.58–3.75)	2.61 (1.62–3.91)	0.10 (0.05–0.14)	0.04 (0.01–0.07)	0.14 (0.12–0.18)
Bangladesh	1.23 (0.78–1.83)	1.07 (0.67–1.59)	1.17 (0.72–1.79)	-0.13 (-0.18 to -0.08)	0.10 (0.04–0.17)	-0.05 (-0.12 to 0.01)
Cambodia	0.75 (0.45–1.16)	0.62 (0.38–0.96)	0.67 (0.40–1.04)	-0.17 (-0.23 to -0.11)	0.07 (0.00-0.16)	-0.11 (-0.18 to -0.04)
Afghanistan	3.74 (2.36–5.54)	3.36 (2.09–5.01)	3.24 (2.02–4.84)	-0.10 (-0.14 to -0.06)	-0.04 (-0.08 to 0.01)	-0.13 (-0.18 to -0.09)
Nepal	0.91 (0.58–1.38)	0.69 (0.43–1.05)	0.88 (0.55–1.33)	-0.24 (-0.29 to -0.19)	0.26 (0.20–0.33)	-0.04 (-0.11 to 0.04)

a: ASR-YLDs, age-standardized years lived with disability; AEMT, adverse effects of medical treatment; SDI, sociodemographic index; ARC, annual rate of change; UI, uncertainty interval

**Table 4:** ASR-YLLs (per 100,000 population) and their relative changes in countries and regions with different SDIs in 1990, 2010 and 2019 due to AEMT.

Region	ASR-YLLs (95% UI)			ACR (95% UI)		
	1990	2010	2019	1990–2010	2010–2019	1990–2019
Global	86.76 (64.85–99.64)	57.33 (46.37–65.17)	48.07 (39.28–55.41)	-0.34 (-0.39 to -0.26)	-0.16 (-0.23 to -0.08)	-0.45 (-0.51 to -0.35)
High SDI	31.58 (27.32–33.79)	23.33 (20.90-25.14)	22.29 (19.44–24.07)	-0.26 (-0.31 to -0.16)	-0.04 (-0.08 to 0.00)	-0.29 (-0.35 to -0.21)
High-middle SDI	44.72 (36.54–49.62)	24.84 (22.58–26.75)	20.71 (17.98–22.70)	-0.44 (-0.50 to -0.35)	-0.17 (-0.22 to -0.11)	-0.54 (-0.59 to -0.45)
Middle SDI	68.19 (51.07–76.89)	34.43 (29.67–39.56)	26.91 (23.38–30.98)	-0.50 (-0.55 to -0.39)	-0.22 (-0.28 to -0.16)	-0.61 (-0.66 to -0.50)
Low-middle SDI	143.32 (103.02-167.35)	91.60 (69.81-102.49)	75.45 (58.92–87.93)	-0.36 (-0.42 to -0.27)	-0.18 (-0.29 to -0.07)	-0.47 (-0.56 to -0.36)
Low SDI	201.97 (141.85-268.46)	137.77 (100.87-192.16)	111.94 (83.89-152.65)	-0.32 (-0.40 to -0.22)	-0.19 (-0.26 to -0.09)	-0.45 (-0.53 to -0.34)
United States	36.48 (32.48–40.21)	27.03 (25.51–32.96)	26.01 (24.44–31.31)	-0.26 (-0.29 to -0.10)	-0.04 (-0.06 to -0.01)	-0.29 (-0.32 to -0.15)
Japan	10.97 (10.38–13.76)	16.49 (12.19–17.37)	15.29 (11.44–16.57)	0.50 (-0.08 to 0.62)	-0.07 (-0.14 to -0.02)	0.39 (-0.10 to 0.55)
Australia	20.24 (18.60-23.67)	22.46 (18.04–24.15)	19.41 (16.18–21.33)	0.11 (-0.10 to 0.22)	-0.14 (-0.20 to -0.05)	-0.04 (-0.19 to 0.07)
Argentina	147.19 (112.62-156.48)	87.62 (80.77-101.27)	79.52 (72.19–92.65)	-0.40 (-0.45 to -0.09)	-0.09 (-0.16 to -0.03)	-0.46 (-0.51 to -0.14)
Malaysia	46.03(32.23–58.58)	25.82 (20.88–29.21)	22.25 (16.67–28.68)	-0.44 (-0.56 to -0.29)	-0.14 (-0.35 to 0.10)	-0.52 (-0.69 to -0.31)
China	66.63 (43.18–80.40)	17.30 (15.31–21.78)	11.31 (9.55–14.73)	-0.74 (-0.80 to -0.58)	-0.35 (-0.43 to -0.26)	-0.83 (-0.87 to -0.72)
India	151.77 (106.99-176.02)	100.32 (74.71-113.59)	80.91 (60.18–97.61)	-0.34 (-0.41 to -0.25)	-0.19 (-0.34 to -0.05)	-0.47 (-0.57 to -0.34)
Russian Federation	21.58 (19.44–30.41)	32.14 (24.92–33.60)	28.24 (20.35–32.87)	0.49 (-0.13 to 0.70)	-0.12 (-0.26 to 0.02)	0.31 (-0.21 to 0.60)
Bangladesh	171.78 (121.34-231.41)	92.61 (67.69-138.19)	65.51 (46.39–96.71)	-0.46 (-0.57 to -0.31)	-0.29 (-0.41 to -0.15)	-0.62 (-0.71 to -0.49)
Cambodia	96.47 (71.42-143.74)	49.99 (34.63–79.15)	39.63 (27.07–62.95)	-0.48 (-0.60 to -0.34)	-0.21 (-0.34 to -0.04)	-0.59 (-0.70 to -0.46)
Afghanistan	341.59 (150.94-525.11)	219.05 (104.89-360.18)	163.07 (81.75-259.34)	-0.36 (-0.51 to -0.16)	-0.26 (-0.38 to -0.09)	-0.52 (-0.64 to -0.34)
Nepal	162.44 (109.31-242.02)	94.73 (67.91-142.96)	84.19 (60.23-122.86)	-0.42 (-0.54 to -0.27)	-0.11 (-0.26 to 0.07)	-0.48 (-0.60 to -0.33)

a: ASR-YLLs, age-standardized years of life lost; AEMT, adverse effects of medical treatment; SDI, sociodemographic index; ARC, annual rate of change; UI, uncertainty interval

The sex ratio (female:male) of ASR-YLDs due to AEMT remained relatively stable from 1990 to 2019 and was positively correlated with the SDI. Globally, women had higher ASR-YLDs for AEMT than men. This was especially true in China, where the female-to-male ratio of ASR-YLDs due to AEMT fluctuated around 1.20. The sex ratio (female:male) of ASR-YLLs for AEMT was more dispersed than that of ASR-YLDs, and the trend was more similar to that of ASR-DALYs due to AEMT. In 2019, the sex ratio (female:male) of ASR-YLLs in low-middle-SDI regions was 1.23, which was the highest among all the SDI regions, while the ratio in high-SDI regions was the lowest, at only 0.76 (Fig. 3B).

The YLD and YLL rates attributed to AEMT in China, middle-SDI regions and worldwide were relatively high in the 0- to 10-year-old age group, with a significant downward trend. Inconsistent with what was observed globally, the AEMT-related YLD rate in China stabilized and decreased among individuals older than 20 years. Moreover, the global AEMT-related YLL rate increased with age in China and among elderly individuals (over 60 years old) in middle-SDI regions and worldwide. The age distribution trend of the YLL rate attributed to AEMT in China was lower than that worldwide and in middle-SDI regions at any age (Fig. 3C).

It is predicted that the ASR-YLLs due to AEMT will continue to decrease worldwide until 2040. The global ASR-YLLs due to AEMT is predicted to reach 54.7 2 (95% UI: 45.24–63.18) by 2030 and 50.21 (95% UI: 40.71–59.29) by 2040. The ASR-YLLs due to AEMT in China will remain at the lower-middle level, estimated to be 13.43 (95% UI: 10.95–18.40) in 2030 and 10.61 (95% UI: 8.24–14.71) in 2040 (Fig. 3D).

### Rates of prevalence and incidence due to AEMT in China, worldwide and in other countries and regions with different SDI levels, 1990–2019

**Fig. 4:**
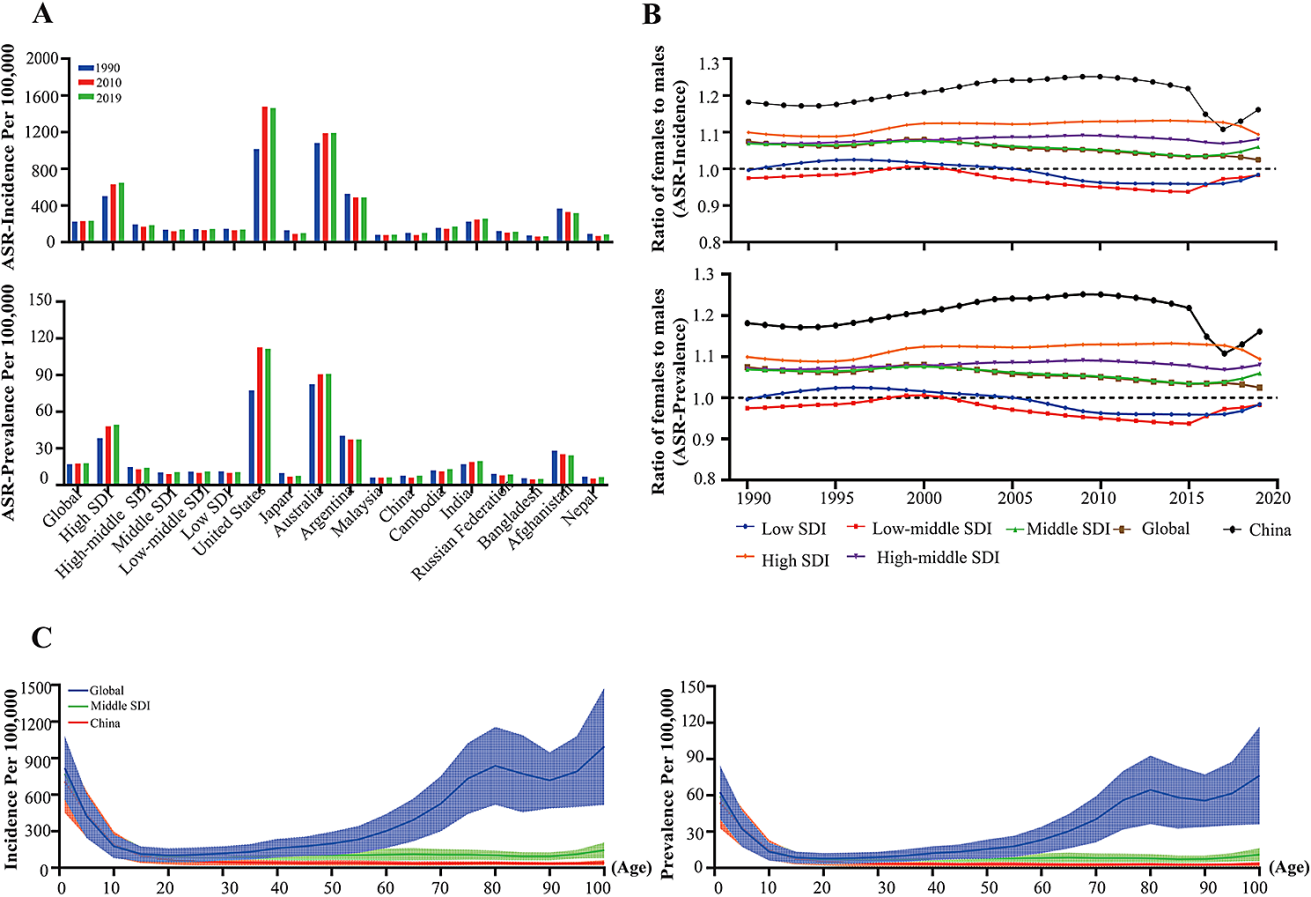
Comparison of the ASR of the incidence and ASR of the prevalence caused by AEMT in China and in different SDI regions from 1990 to 2019. **(A) **Comparison of the incidence and prevalence of ASR in China and SDI regions and globally in 1990, 2010 and 2019. **(B)** Sex differences and trends in ASR incidence and ASR prevalence of AEMT from 1990 to 2019. **(C)** Age differences in ASR incidence and prevalence due to ASR caused by AEMT between China and the SDI region

The incidence of ASR due to AEMT showed an upward trend in high-SDI regions, with an ARC of 0.29 (95% UI: 0.24–0.35). During this period, the ASR-incidence attributed to AEMT in China decreased first, from 100.18 (95% UI: 75.53-129.64) in 1990 to 78.81 (95% UI: 57.33-103.66) in 2010, and then rebounded to 100.30 (95% UI: 72.66-133.22) in 2019 (Table 5; Fig. 4A). The female-to-male ratios of the ASR of prevalence and ASR of incidence due to AEMT did not change significantly between 1990 and 2019 across SDI regions. There was a positive correlation between the sex ratio of the ASR of the incidence (female:male) and the SDI. In China, the ASR of the incidence and ASR of the prevalence of AEMT among females were greater than those among males (Fig. 4B). As shown in Fig. 4C, the ASR of the incidence of AEMT in 2019 was high among people younger than 20 years, decreased with age, remained at a very low level between 20 and 50 years of age, and increased among those 50 years and older; moreover, the overall trend was not affected, although there was a slight decrease in the 80–90 age group.

**Table 5:** ASRs of the incidence (per 100,000 population) and their relative changes due to AEMT in countries and regions with different SDI levels in 1990, 2010 and 2019

Region	ASR-incidence (95% UI)			ARC (95% UI)		
	1990	2010	2019	1990–2010	2010–2019	1990–2019
Global	223.56 (186.25–265.00)	230.43 (196.18–268.60)	233.31 (193.56-277.63)	0.03 (0.00-0.07)	0.01 (-0.02 to 0.05)	0.04 (0.02–0.07)
High SDI	501.76 (416.54-603.71)	630.09 (538.63-735.22)	647.57 (543.25-769.87)	0.26 (0.17–0.34)	0.03 (-0.01 to 0.07)	0.29 (0.24–0.35)
High-middle SDI	194.20 (156.75-238.76)	168.40 (137.24-205.09)	185.13 (146.49-228.55)	-0.13 (-0.15 to -0.12)	0.10 (0.06–0.13)	-0.05 (-0.07 to -0.02)
Middle SDI	136.02 (108.89-167.77)	119.45 (96.28-147.57)	138.27 (109.31-172.43)	-0.14 (-0.15 to -0.13)	0.16 (0.13–0.19)	0.00 (-0.03 to 0.03)
Low-middle SDI	143.17 (118.47-170.08)	130.52 (107.53-153.51)	145.43 (118.09-175.65)	-0.10 (-0.11 to -0.08)	0.11(0.08–0.15)	0.00 (-0.03 to 0.04)
Low SDI	146.68 (122.45-172.09)	130.11 (108.90-152.41)	138.40 (114.21-164.47)	-0.15 (-0.17 to -0.14)	0.06(0.03–0.09)	-0.10 (-0.13 to -0.08)
United States	1014.55 (849.15-1211.26)	1477.51 (1252.50-1739.77)	1461.98 (1217.60-1743.21)	0.46 (0.33–0.58)	-0.01(-0.06 to 0.04)	0.44 (0.36–0.54)
Japan	129.15 (99.97-162.64)	91.16 (76.50-108.05)	99.75 (81.04-120.34)	-0.29 (-0.36 to -0.21)	0.09 (0.04–0.14)	-0.23 (-0.28 to -0.17)
Australia	1081.33 (893.85-1287.27)	1187.90 (997.61-1408.21)	1190.71 (988.26-1416.48)	0.10 (0.04 to 0.16)	0.00 (-0.05 to 0.05)	0.10 (0.05–0.15)
Argentina	528.30 (455.70-612.55)	488.57 (419.79-563.18)	488.64 (413.08-577.26)	-0.08 (-0.1 to -0.02)	0.00 (-0.05 to 0.06)	-0.08 (-0.13 to -0.02)
Malaysia	80.38 (61.93-102.54)	78.38 (58.95–101.20)	82.80 (61.84-111.04)	-0.02 (-0.08 to 0.03)	0.06 (-0.01 to 0.13)	0.03 (-0.04 to 0.12)
China	100.18 (75.53-129.64)	78.81 (57.33-103.66)	100.30 (72.66-133.22)	-0.21 (-0.25 to -0.18)	0.27 (0.23–0.32)	0.00 (-0.06 to 0.07)
India	156.78 (129.22–185.90)	147.24 (120.92–173.20)	170.90 (138.66-206.58)	-0.06 (-0.08 to -0.04)	0.16 (0.12–0.20)	0.09 (0.05–0.14)
Russian Federation	224.09 (179.06-278.15)	246.16 (201.06-297.23)	256.14 (207.49–313.00)	0.10 (0.05–0.14)	0.04 (0.01–0.07)	0.14 (0.12–0.17)
Bangladesh	120.77 (101.02-143.04)	104.55 (86.83-123.43)	114.53 (93.48-139.01)	-0.13 (-0.18 to -0.08)	0.10 (0.04–0.17)	-0.05 (-0.11 to 0.01)
Cambodia	73.39 (57.80–91.60)	61.26 (47.62–78.11)	65.35 (49.81–83.73)	-0.17 (-0.23 to -0.11)	0.07 (0.00-0.16)	-0.11 (-0.18 to -0.04)
Afghanistan	367.35 (311.38-425.84)	329.38 (282.42-380.25)	317.78 (268.01-374.77)	-0.10 (-0.14 to -0.06)	-0.04 (-0.08 to 0.01)	-0.13 (-0.18 to -0.09)
Nepal	89.76 (75.53-105.99)	68.22 (56.67–82.03)	86.28 (70.49-104.65)	-0.24 (-0.29 to -0.19)	0.26 (0.20–0.33)	-0.04 (-0.11 to 0.04)

a: ASR- incidence, age-standardized incidence rate; AEMT, adverse effects of medical treatment; SDI, sociodemographic index; ARC, annual rate of change; UI, uncertainty interval

## Discussion

At present, the reporting and management of AEMT in China are still in their infancy. The findings show that the main categories of medical adverse events in China include errors in information delivery, medication administration, and program management. In addition, there are incidents related to care and equipment use [18]. The largest decrease in China’s ASDR attributed to AEMT was from 1990 to 2019. Moreover, during this period, both China’s ASR-DALYs and ASR-YLLs significantly decreased. These improvements may have arisen from the adjustment and development of public health in China during this period. From 1990 to 2010, the government vigorously strengthened the network construction of rural medical and health care by increasing the investment in medical and health funds, expanding the supply of medical and health resources, and improving medical and health institutions [19]. A series of measures, such as establishing rural clinics and township hospitals, training rural doctors and implementing rural medical programs, greatly improved the medical status of rural residents in China and ensured that villagers received timely and effective treatment when they are sick or injured [20, 21]. However, most countries aim to reduce mortality due to clinical accidents but ignore the burden of complications and disability associated with AEMT [22, 23]. Therefore, the change in ASR-YLDs in China was not obvious, which highlights the challenges in meeting the health needs of economically developing countries in rapid economic transition.

From 1990 to 2019, the global ASR of the incidence of AEMT exhibited a fluctuating upward trend. Notably, areas with high SDIs had higher morbidity rates and lower mortality rates. This is related to many factors, such as countries and regions with high SDIs having better basic medical systems and more standardized adverse event reporting systems; additionally, medical staff are paying more attention to AEMT, and the public has a deeper understanding of AEMT. The presence of these factors enables high-SDI regions to accurately record the occurrence of AEMT in a timely manner and learn from them to avoid similar incidents.

In contrast to regions with high SDI levels, other SDI regions exhibited a downward then upward trend, as did China. Due to the lack of a complete reporting system in rural medical models, the statistics of the incidence of AEMT are overdependent on the personal behaviors of medical staff [24]. This further leads to large errors and uncertainties in the statistics of AEMT, which are possible reasons why the incidence rate of AEMT decreased dramatically between 1990 and 2010. From 2010 to 2019, the Chinese government paid more attention to national health issues and proposed a new reform plan for the medical and health system in 2009. Through this reform, a four-in-one basic medical and health security system covering the entire population was initially established; this system consists of a medical service system, a public health service system, a drug supply security system and a medical security system [25, 26]. Moreover, China has strengthened its relationships with the WHO to promote international medical cooperation. During this period, China gradually formed a reporting system for AEMT that combined a compulsory reporting system, voluntary reporting system and internal reporting system. In addition, big data play a nonnegligible role in medical adverse event reporting. For example, the use of electronic medical records and artificial intelligence (AI) has led to more complete statistics on adverse medical events among patients [27–30]. Second, China’s basic medical insurance system was implemented in the early 21st century and has developed rapidly since then. By 2012, China’s rural cooperative medical care coverage had reached 95%. Other medical security systems for urban workers are also making steady progress under the supervision of the government [31]. Finally, with the increase in national economic income and the strengthening of people’s understanding of commercial insurance, an increasing number of people recognize and purchase commercial medical insurance [32, 33]. These factors reduce the ASDR and ASR-DALYs of AEMT but increase the ASR of the incidence of AEMT.

In terms of sex, the ASR of the incidence and prevalence of AEMT among Chinese women were greater than those among men, while the ASDR of AEMT among women was lower than that among men. The female-to-male ratios of ASR-DALYs and ASR-YLLs changed greatly in China during 1990–2019. The high ASR of the incidence and ASR of the prevalence of AEMT among women was because women are more susceptible to adverse drug reactions than men are, and women have a greater probability of accidents during surgery [34]. Some women tend to fall into a negative state after illness. Because men and women are biologically different, women have a greater risk burden during pregnancy and childbirth [35]. Many men are engaged in heavy physical work, and their average life expectancy is shortened. Moreover, smoking, drinking and other unhealthy habits are associated with greater mortality among males than among females [36–38]. Since the beginning of the 21st century, the social status of Chinese women has gradually improved, and an increasing number of women have become economically independent. The impact of treatment and rehabilitation burden after accidental injury has also been reduced, which has a great impact on the follow-up treatment of patients. As a result, the ASR-DALYs of Chinese men increased during the observation period.

The incidence of AEMT was highest among children (0–10 years old) in China because their bodies are not mature enough to adapt to the outside world, and their resistance to disease is weak. Surgical and perioperative adverse events, drug dose effects and accidents are the main causes of high mortality [39]. In addition, patients older than 65 years have reduced physical function and a reduced ability to recover after surgery and are more prone to multiple complications, which greatly limits the effectiveness of medications and treatments for older patients [40, 41]. Multiple morbidities is a major challenge facing global health care systems, and the current GBD often fails to account for the co-occurrence of multiple diseases, thus increasing the uncertainty of AEMT data.

Our research has important implications for global health care delivery. This study can not only increase the public’s attention regarding AEMT but also help the government fully utilize its administrative functions, formulate relevant policies and laws, and promote the unified management of an AEMT reporting system. This approach is also conducive to training medical staff on AEMT so that they can respond to adverse events in hospitals in a timely and efficient manner. However, AEMT still faces many problems and challenges, such as how to effectively reduce the occurrence of AMET and how to reduce the harm caused by AEMT as soon as possible.

Based on GBD data and China’s actual development experience, we summarize the concerns and challenges of addressing the AEMT burden in China from 1990 to 2019. This research has certain limitations. First, the data relied on secondary information obtained from the GBD study rather than from the clinical setting. Second, each country and region have different criteria for classifying adverse events, and these factors may fundamentally affect our results. Third, the basic public facilities in some economically underdeveloped areas are not perfect, and the reporting consciousness of medical staff is not sound, resulting in large errors in the statistical data. Finally, the cause classification system of the GBD study classifies each death as a single root cause, which can lead to some events related to AEMT that may be classified elsewhere.

## Conclusions

From 1990 to 2019, the world experienced rapid social and economic changes, medical conditions in countries and regions greatly improved, and medical systems gradually improved. Much progress has been made globally in reducing the ASDRs and ASR-DALYs caused by AEMT, but some countries with rapidly changing economies have yet to achieve effective results in reducing the incidence of AEMT. The same is true of China. This requires countries to continuously strengthen the reform of the AEMT reporting system and governments and relevant personnel to pay more attention to AEMT and take effective measures to reduce the occurrence of AEMT.

## Data Availability

The datasets used and/or analyzed during the current study are available from the corresponding author upon reasonable request.

## Abbreviations

AEMT: adverse effects of medical treatment
ARC: annual rate of change
ASDR: age-standardized death rate
ASR: age-standardized rate
DALYs: disability-adjusted life years
YLDs: years lived with disability
YLLs: years of life lost
GBD: Global Burden of Disease
ICD: International Classification of Diseases
SDI: sociodemographic index
UI: uncertainty interval
WHO: World Health Organization
